# Dexmedetomidine in Spinal Anesthesia for Orthopedic Surgery: Extending Analgesia Beyond the Operating Room

**DOI:** 10.7759/cureus.95136

**Published:** 2025-10-22

**Authors:** Rafail Ioannidis, Christos Zapantis-Gakis, Theodora Zarogianni, Neoklitsa Manouskou, Despoina Sarridou

**Affiliations:** 1 Anesthesiology and Pain Medicine, General Hospital of Drama, Drama, GRC; 2 Anesthesiology and Intensive Care, AHEPA University Hospital of Thessaloniki, Thessaloniki, GRC

**Keywords:** adjuvant, dexmedetomidine, prolonged analgesia, spinal anesthesia, total knee arthroplasty

## Abstract

Spinal anesthesia is widely used in total knee arthroplasty (TKA) because it provides reliable block characteristics and favorable perioperative outcomes. However, a key limitation is the relatively short duration of local anesthetics, which may require additional analgesia or a switch to general anesthesia. To address this, intrathecal adjuvants are used to extend the duration of the block and enhance postoperative pain management. Dexmedetomidine, a highly selective α₂-adrenergic agonist, has demonstrated sedative and analgesic properties without respiratory depression, making it an attractive alternative to opioids such as fentanyl. In this case series, 10 µg of intrathecal dexmedetomidine was combined with ropivacaine in 20 patients undergoing TKA. Outcomes assessed included onset and regression of sensory and motor block, postoperative pain scores, and hemodynamic stability.

Dexmedetomidine produced a rapid block onset, extended sensory block lasting up to 12 hours in some patients, and a predictable pattern of motor regression. Postoperative analgesia was favorable, with most patients remaining pain-free for the first six hours and reporting only mild discomfort at 12 hours. Hemodynamic changes observed included reductions in heart rate (HR) and mean arterial pressure (MAP), with some patients requiring vasopressor support, though no serious adverse events occurred.

Our findings align with randomized controlled trials (RCTs) and meta-analyses indicating that intrathecal dexmedetomidine extends block duration, reduces the need for postoperative analgesics, and offers a favorable efficacy and side-effect profile compared to fentanyl. This case series adds to the existing body of evidence by presenting real-world clinical data on the use of intrathecal dexmedetomidine as an adjuvant to ropivacaine in elderly patients undergoing TKA. It demonstrates the adjuvant’s safety, hemodynamic stability, and analgesic effectiveness in a routine hospital setting, supporting the applicability of findings from controlled trials to everyday anesthetic practice. Further large-scale studies are needed to refine dosing strategies and evaluate long-term outcomes in orthopedic populations.

## Introduction

Spinal anesthesia is commonly used for lower-limb surgeries such as total knee arthroplasty (TKA), hip replacement, and fracture fixation due to its rapid onset, reliable and dense neural blockade, high success rates, and minimal need for airway manipulation. Compared with general anesthesia, it is associated with reduced systemic drug exposure, less intraoperative blood loss, decreased risk of thromboembolic events, and improved hemodynamic stability in appropriately selected patients [1,2]. Moreover, spinal anesthesia is frequently associated with improved postoperative pain control during the immediate recovery period, a lower incidence of postoperative nausea and vomiting, and earlier mobilization, factors that collectively support the goals of Enhanced Recovery After Surgery (ERAS) protocols [3].

Despite these advantages, one of the major limitations of spinal anesthesia is the relatively short duration of action of commonly used local anesthetics. This can be problematic in lengthy orthopedic procedures such as TKA, where prolonged analgesia is often required not only intraoperatively but also in the early postoperative period [4]. Inadequate duration of analgesia may necessitate conversion to general anesthesia intraoperatively or the use of systemic opioids postoperatively, both of which can compromise recovery and increase the risk of complications [5]. To overcome this challenge, intrathecal adjuvants - including opioids (e.g., fentanyl, morphine), α₂ adrenergic agonists (e.g., clonidine, dexmedetomidine), and other agents - are frequently combined with local anesthetics. These adjuvants have been shown to prolong the duration of sensory and motor block, improve intraoperative anesthesia quality, and extend postoperative analgesia without significantly increasing side effects [6-8].

Fentanyl, a lipophilic μ-opioid receptor agonist, has been the most common opioid adjuvant, offering rapid onset and effective intraoperative analgesia. Yet it is associated with adverse effects such as pruritus, nausea, urinary retention, and the risk of respiratory depression [8]. Dexmedetomidine, a highly selective α₂-adrenergic agonist, provides sedation, anxiolysis, and analgesia without respiratory depression [9]. When used intrathecally, it inhibits nociceptive transmission by reducing substance P and glutamate release in the dorsal horn and hyperpolarizing postsynaptic neurons [10]. Importantly, it prolongs sensory and motor block and improves postoperative analgesia compared with fentanyl, and it plays a very important role in every opioid-free anesthesia (OFA) protocol [11,12].

Several randomized controlled trials (RCTs) and meta-analyses have demonstrated that intrathecal dexmedetomidine produces significantly longer sensory and motor block durations and reduces the need for rescue analgesics, with a lower incidence of pruritus compared to fentanyl [11-13]. Preclinical neurotoxicity data are limited but reassuring: several animal histologic and electron-microscopic studies found no consistent evidence of neuronal injury after single-dose intrathecal dexmedetomidine at doses used in clinical trials. However, intrathecal use remains off-label in many regions, and long-term safety data are limited; therefore, cautious application and awareness of local regulatory guidelines are essential [14]. Dosing in published human studies is heterogeneous but clusters around 5-10 µg intrathecal [15,16]. These findings are particularly relevant in TKA, a surgery associated with severe early postoperative pain.

## Materials and methods

Ethics approval for this study was granted by the Board of Directors of General Hospital of Drama, Greece, under registration number 155/2024, dated April 8, 2024. This descriptive observational case series study included 20 orthopedic patients who presented to the hospital for a TKA surgery, focusing on clinical implications and advantages of dexmedetomidine as an intrathecal adjuvant to local anesthetic. All participants provided informed consent, including the hospital’s standard consent form for anesthesia and surgical procedures. Participants were fully informed about the study objectives and procedures.

The primary objectives of the study were to present a series of cases in which dexmedetomidine was used as an adjuvant to ropivacaine in spinal anesthesia for TKA and, based on the clinical data obtained, to further support the existing literature. The secondary objectives were to evaluate the safety profile of dexmedetomidine in spinal anesthesia with respect to the anesthetic efficacy and prolongation of analgesia, its pharmacokinetic, pharmacodynamic parameters, and the clinical investigation of possible side effects. Exclusion criteria included patients requiring general anesthesia due to medical contraindications to spinal anesthesia or personal refusal to undergo spinal anesthesia, as well as patients with a preoperative baseline heart rate (HR) of fewer than 50 beats per minute (bpm), given the risk of further exacerbation of bradycardia associated with dexmedetomidine.

The study population consisted of 20 patients with a mean age of 73.2 years (range: 56-84 years). The study included 10 females and 10 males, providing a balanced representation of both sexes. All patients, classified as American Society of Anesthesiologists (ASA) physical status I-III, received spinal anesthesia at the L3-L4 interspace using a 25-gauge pencil-point needle, also known as an atraumatic spinal needle. Each patient received around 20 mg of ropivacaine (2.7 ml of isobaric ropivacaine 0.75%) combined with 10 µg of dexmedetomidine.

Variables recorded included HR and mean arterial pressure (MAP) before and after spinal injection; intraoperative vasopressor requirements (e.g. ephedrine or phenylephrine); sensory block level at 30 seconds, one minute and two, six, and 12 hours post-spinal; motor block assessed with Bromage scale; pain intensity measured with visual analog Scale (VAS) preoperatively and at two, six, and 12 hours postoperatively [17]. The Bromage scale is a clinical tool used to evaluate the level of motor block following regional anesthesia, particularly spinal or epidural. It usually ranges from 0 to 3, with 0 indicating full movement of the legs and feet, and 3 representing a complete inability to move the legs or feet. The VAS score is a tool used to measure a patient’s subjective level of pain intensity on a continuous scale, usually from 0 (no pain) to 10 (worst imaginable pain) [18]. This was a descriptive observational case series. Data were analyzed qualitatively to highlight trends.

Statistical analysis

Data were analyzed using descriptive statistics. Continuous variables (e.g., heart rate, mean arterial pressure, VAS scores) were assessed for distribution and reported as mean ± standard deviation (SD) when approximately normally distributed or as median (range) when non-normally distributed. Ordinal variables (e.g., Bromage motor block scores, sensory block levels) were summarized as frequencies and percentages at each observation time point. The requirement for vasoactive agents (ephedrine, phenylephrine) was expressed as absolute frequencies and total administered doses. As this is a case series, no inferential statistical tests were performed; results are presented descriptively to illustrate observed clinical patterns. All patients completed the predefined assessments with no missing data. Statistical analyses were performed using Microsoft Excel 2021 (Microsoft Corporation, Redmond, WA) and IBM SPSS Statistics version 28 (IBM Corp., Armonk, NY).

## Results

**Figure 1:**
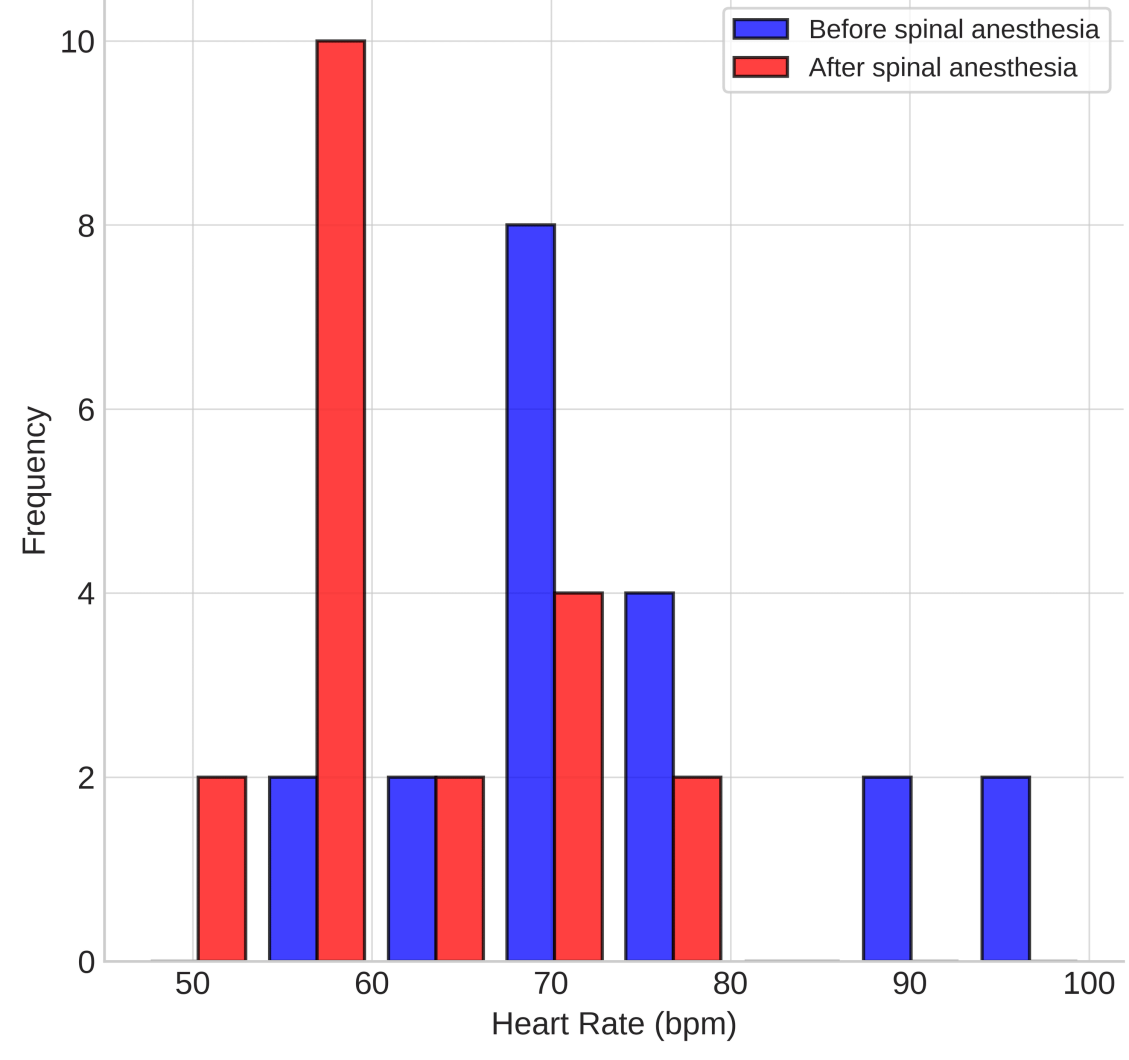
Heart rate (HR) distribution

**Figure 2:**
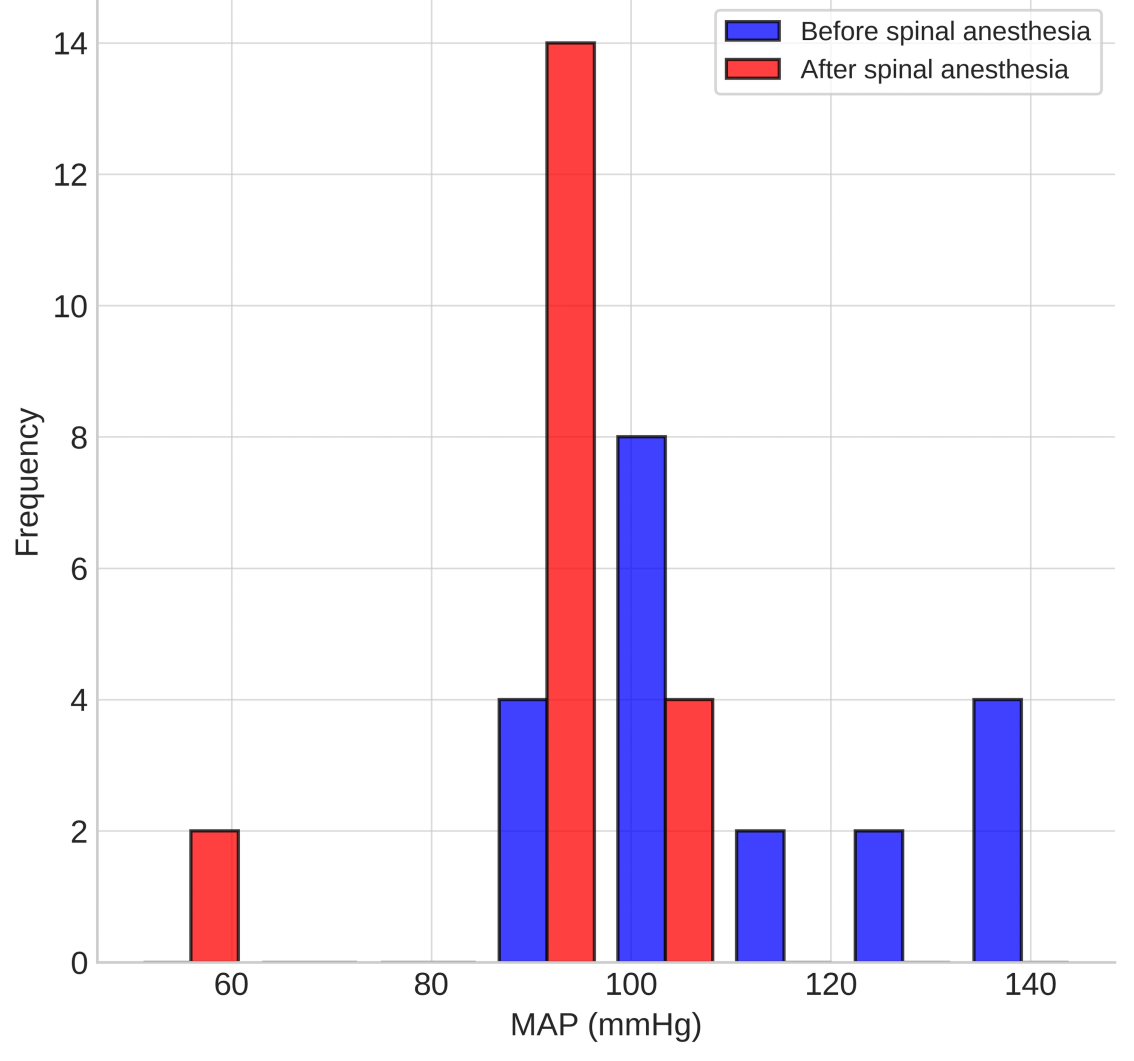
Mean arterial pressure (MAP) distribution

The data analysis was as follows: hemodynamics (Figures 1, 2): baseline HR ranged from 60 to 100 bpm, decreasing after spinal anesthesia to 47-75 bpm. MAP dropped from pre-spinal values of 89-145 mmHg to post-spinal values of 50-103 mmHg. Ephedrine was required in six patients, specifically 10-30 mg total, and phenylephrine in two, specifically 0.7 mg and 0.1 mg.

Sensory block-related data are provided in Table 1. At 30 seconds post-spinal, the predominant sensory block was observed at L1 (n=8, 40%) and T10 (n=6, 30%), indicating that the initial spread of anesthesia was primarily limited to the lower thoracic and upper lumbar dermatomes. Minor sensory involvement was noted at L2 (n=4, 20%) and L4 (n=2, 10%), with no higher thoracic levels (T6-T8) affected at this time point. At one minute post-spinal, the block ascended to higher dermatomes. The most common sensory level was T10 (n=10, 50%), followed by T12 (n=4, 20%) and T6, T7, and T8 (n=2 each, 10%). Lumbar levels (L1-L4) showed no remaining dominant sensory block at this time. At two hours postoperatively, the sensory block remained at mid-thoracic levels, with the majority at T10 (n=8, 40%), T12 (n=4, 20%), and T7-T8 (n=2 each, 10%). Residual block was still present at L1 (n=2, 10%), while no level of block was reported in two patients (10%). At six hours postoperatively, a clear regression of sensory anesthesia was observed. The highest concentration of residual block was noted at L1 (n=6, 30%), while smaller proportions remained at L3 (n=2, 10%), L4 (n=4, 20%), and L5 (n=2, 10%). No level of sensory block was present in six patients (30%), confirming significant recovery. At 12 hours postoperatively, complete regression of the sensory block was documented in the majority of patients (no level, n=16, 80%). Minimal residual block persisted at L3 and L4 (n=2 each, 10%).

**Table 1:** Sensory block level recorded at each of the four time points Sensory block height refers to the highest dermatome level at which a patient loses sensation following spinal anesthesia. It is assessed using methods such as pinprick testing or cold sensation and provides a measure of how far the anesthetic agent has spread within the subarachnoid space.

Level	30 seconds post-spinal	One minute post-spinal	Two hours postoperative	Six hours postoperative	12 hours postoperative
T6	0	2	0	0	0
T7	0	2	2	0	0
T8	0	2	2	0	0
T10	6	10	8	0	0
T12	0	4	4	0	0
L1	8	0	2	6	0
L2	4	0	0	0	0
L3	0	0	0	2	2
L4	2	0	0	4	2
L5	0	0	0	2	0
No level	0	0	2	6	16

The data related to the motor block are summarized in Table 2. The Bromage scale, expressed using Roman numerals as grades I-IV, corresponds to the numeric scale 0-3. At 30 seconds post-spinal, the majority of patients exhibited Grade III (n=14, 70%) and Grade IV (n=6, 30%) motor block, indicating a rapid onset of moderate to complete lower limb paralysis. No patients remained in Grade I or II (n=0). At one minute post-spinal, the most common grade was Grade II (n=10, 50%), followed by Grade III (n=6, 30%) and Grade I (n=4, 20%). This early variation in motor response may reflect individual pharmacodynamic variability or differences in drug spread. At two hours postoperatively, the majority of patients had regained full motor function, with Grade I (n=18, 90%) being predominant. A small minority remained with Grade IV motor block (n=2, 10%), while Grades II and III were completely absent (n=0). At six hours postoperatively, motor block distribution became more varied. An equal proportion of patients were classified as Grade I (n=6, 30%) and Grade IV (n=6, 30%), while Grades II and III were each observed in four patients (20%). At 12 hours postoperatively, a significant number of patients remained in Grade IV (n=14, 70%), indicating persistent and complete motor block well beyond the expected duration. The remaining patients were distributed equally across Grades I, II, and III (n=2 each, 10%).

**Table 2:** Bromage scale at each of the four time points The Bromage scale is a clinical tool used to assess the degree of motor block after regional (especially spinal or epidural) anesthesia. It typically ranges from 0 to 3, where: 0 = Full movement of legs and feet, 3 = Complete inability to move legs or feet. In the table, the scale is expressed using Roman numerals as grades I–IV, corresponds to the numeric scale 0–3.

Grade	30 seconds post-spinal	One minute post-spinal	Two hours postoperative	Six hours postoperative	12 hours postoperative
I	0	4	18	6	2
II	0	10	0	4	2
III	14	6	0	4	2
IV	6	0	2	6	14

The data pertaining to pain are shown in Table 3. At preoperative assessment, the mean VAS score was 4.1, with a standard deviation (SD) of 2.27, indicating a moderate level of pain reported by patients before spinal anesthesia. The pain scores ranged from a minimum of 0.5 to a maximum of 7.5, reflecting variability in baseline pain perception across the sample. At two hours postoperatively, the mean VAS score dropped significantly to 0.2, with a low SD of 0.62. Scores ranged from 0 to 2, demonstrating effective analgesia in the immediate postoperative period. The near-zero mean and narrow range suggest that most patients were nearly pain-free at this time, consistent with the peak efficacy of spinal anesthesia. At six hours postoperatively, the mean VAS score increased to 1.7, accompanied by a higher SD of 2.91. The pain scores ranged from 0 to 8.5, suggesting that while some patients remained pain-free, others began experiencing moderate to severe discomfort. This distribution reflects the expected wearing off of spinal anesthesia and the transition to systemic analgesic coverage. At 12 hours postoperatively, the mean VAS score rose further to 4.6, exceeding the preoperative mean and accompanied by an SD of 3.12. Reported pain ranged from 0.5 to 8.5, indicating significant variability and a notable return of pain in many patients.

**Table 3:** VAS scores at each of the four time points The VAS score is a tool used to measure a patient’s subjective level of pain intensity on a continuous scale, usually from 0 (no pain) to 10 (worst imaginable pain) VAS: visual analog scale; SD: standard deviation

Time point	Mean	SD	Minimun	Maximum
Preoperative	4.1	2.268781	0.5	7.5
Two hours postoperative	0.2	0.615587	0	2
Six hours postoperative	1.7	2.912767	0	8.5
12 hours postoperative	4.6	3.118704	0.5	8.5

In this study, the use of intrathecal dexmedetomidine as an adjuvant to spinal anesthesia in patients undergoing TKA was associated with a rapid onset and extensive spread of both sensory and motor block. Hemodynamic parameters remained generally stable, though modest reductions in HR and MAP were observed, requiring minimal vasopressor support. Postoperative analgesia was excellent during the early hours, with pain control remaining highly effective for several hours after surgery. A tendency toward prolonged motor block was noted in a small subset of patients, which warrants further evaluation in larger studies.

## Discussion

The prolonged postoperative analgesia and improved block characteristics observed in this series support the growing body of evidence favoring dexmedetomidine as an intrathecal adjuvant over fentanyl in spinal anesthesia, particularly for lower-limb procedures such as TKA. In this patient cohort, the addition of 10 µg intrathecal dexmedetomidine to ropivacaine resulted in a rapid onset of sensory and motor block, extended sensory block levels persisting up to 12 hours in some patients, and favorable pain scores through the early postoperative period.

Multiple RCTs and meta-analyses corroborate these findings. For instance, a systematic review and meta-analysis found that intrathecal dexmedetomidine significantly prolonged the duration of both sensory and motor block, provided a longer pain-free period, and reduced the need for rescue analgesics compared with fentanyl as an adjuvant [6]. Rahimzadeh et al. studied 90 patients undergoing lower limb surgery, using 5 µg dexmedetomidine versus 25 µg fentanyl plus bupivacaine; the dexmedetomidine group demonstrated significantly prolonged durations of both motor and sensory block, extended time before the first request for analgesia, and lower pain scores at six hours postoperatively [19]. Another recent meta-analysis focusing on intrathecal ropivacaine adjuvants reported that dexmedetomidine had more favorable effects versus fentanyl in terms of block duration and analgesic profile [20,21].

Mechanistic differences help explain the superiority of dexmedetomidine in many studies. While fentanyl acts via μ-opioid receptor activation, leading to inhibition of nociceptive transmission, it is limited by its side effects, like pruritus, nausea, urinary retention, and risk (though small at low doses) of respiratory depression. Dexmedetomidine, by contrast, is a highly selective α₂-adrenergic agonist. Intrathecally, it reduces presynaptic release of excitatory neurotransmitters (such as substance P and glutamate) and hyperpolarizes postsynaptic dorsal horn neurons, enhancing and prolonging both sensory and motor blocks with fewer opioid-related adverse events [22]. This mechanism likely accounts for both the longer duration seen in our series (12 hours residual in some patients) and for the lower pain scores with fewer rescue requirements, compared to what is often seen with fentanyl adjuncts.

The hemodynamic changes observed in our study (reduced HR and MAP after spinal anesthesia) are consistent with previous literature on combined spinal with the use of dexmedetomidine. The risk of bradycardia is well described. However, through appropriate patient selection (excluding those with baseline HR <50 bpm) and careful monitoring, the episodes necessitating vasoactive support (six patients with ephedrine, two with phenylephrine) were manageable and did not lead to serious adverse outcomes. This aligns with findings in the study by Rahimzadeh et al., in which hypotension and bradycardia were more common in the dexmedetomidine group but were clinically tolerable [19].

The motor block and regression pattern in our series show that while dexmedetomidine prolongs motor block, the regression is predictable. By two hours, many patients had improved in terms of Bromage scoring, and by 6-12 hours, residual motor block persisted in only a subset. This is clinically relevant: the anesthetic benefit from an extended motor block must be balanced with the need for early mobility, especially after TKA. Our findings suggest that 10 µg dexmedetomidine achieves this balance: enhanced analgesia without excessively delayed motor recovery. Pain control results in our case series are particularly noteworthy. Virtually all patients were pain-free at two hours postoperatively, most remained pain-free at six hours, and although VAS scores rose modestly by 12 hours, these remained lower than often reported in TKA without adjuvant use. This is consistent with meta-analytic findings, which show not only longer block duration but also a longer interval to first rescue analgesic in dexmedetomidine vs. fentanyl groups [20,22].

OFA has emerged as an alternative strategy to mitigate opioid-related adverse effects such as respiratory depression, nausea, ileus, and hyperalgesia. Dexmedetomidine plays a pivotal role in OFA protocols due to its sedative, anxiolytic, and analgesic properties without significant respiratory compromise [23,24]. When administered as an adjuvant to general anesthesia, dexmedetomidine reduces intraoperative opioid requirements, attenuates hemodynamic responses to surgical stress, and prolongs postoperative analgesia [25]. Several RCTs and meta-analyses have demonstrated that intraoperative dexmedetomidine provides superior analgesia and decreased postoperative opioid consumption compared with standard opioid-based regimens [25,26].

Although RCTs and meta-analyses have established the efficacy of intrathecal dexmedetomidine in prolonging sensory and motor block and reducing postoperative analgesic requirements, most of these studies were conducted under tightly controlled conditions that may not fully reflect routine clinical practice. The present case series adds to the existing literature by documenting real-world experience with dexmedetomidine as an adjuvant in elderly orthopedic patients undergoing TKA within a regional hospital setting. These observations highlight the practical feasibility, safety profile, and anesthetic effectiveness of this approach in a population frequently characterized by cardiovascular comorbidities and increased anesthetic risk. Overall, the findings from this case series complement previous trial evidence by supporting its applicability in everyday perioperative care.

Nevertheless, there are certain limitations to both the data of this case series and the published literature. Many RCTs use bupivacaine rather than ropivacaine or different concentrations, which may influence block onset, block height, duration, and side-effect profiles. Dosing is heterogeneous: our dose (10 µg) is on the higher side of what is commonly reported (often 5-10 µg), which may explain longer residual blocks. The off-label nature of intrathecal dexmedetomidine in many jurisdictions remains a concern; long-term neurotoxicity-related data in humans are limited despite reassuring animal studies. Also, large, high-powered head-to-head trials comparing dexmedetomidine versus fentanyl specifically in TKA populations are scarce. Furthermore, it should be noted that this is a single-centre, non-randomized case series study with a small sample size.

We recommend future randomized trials with precise dose-response, with a larger study group, particularly to determine the minimum effective dose of intrathecal dexmedetomidine that optimizes the balance between block duration, side effects, and motor recovery. Comparative trials including significant numbers of frail elderly patients, or those with comorbidities, would be valuable. Moreover, studies that include functional outcomes such as time to mobilization, length of hospital stay, opioid consumption over 24-48 hours, and patient satisfaction will help establish the real-world clinical relevance of dexmedetomidine’s advantages over fentanyl [27]. In addition, it would be very interesting to see if the use of systemic adjuvant dexmedetomidine in conjunction with intrathecal ropivacaine would produce similar results to intrathecal ropivacaine and intrathecal dexmedetomidine.

## Conclusions

The findings of this observational case series, supported by existing clinical evidence, indicate that intrathecal dexmedetomidine is an effective adjuvant to ropivacaine in spinal anesthesia for TKA, offering rapid onset, prolonged sensory and motor block, and superior postoperative analgesia compared with traditional opioid-based regimens. While hemodynamic changes such as bradycardia and hypotension remain relevant considerations, these effects were manageable with careful patient selection and intraoperative monitoring. Importantly, dexmedetomidine’s favorable analgesic profile, reduced opioid-related side effects, and compatibility with opioid-free anesthesia protocols highlight its value in enhancing recovery after surgery. However, additional large-scale, dose-optimization trials focused specifically on TKA populations are necessary to confirm long-term safety, fine-tune dosing regimens, and evaluate functional outcomes such as mobilization and length of hospital stay, thereby helping to establish its role as a preferred intrathecal adjuvant.
